# Pulmonary Localization Revealing Wegener's Granulomatosis

**DOI:** 10.1100/tsw.2010.84

**Published:** 2010-05-04

**Authors:** Mona Mlika, Aida Ayadi-Kaddour, Adel Marghli, Tarek Kilani, Faouzi El Mezni

**Affiliations:** ^1^ Department of Pathology Abderrahman Mami Hospital, Ariana, Tunisia; ^2^ Department of Thoracic Surgery, Abderrahman Mami Hospital, Ariana, Tunisia

**Keywords:** immunosuppressive therapy, pulmonary masses, treatment, vasculitides, Wegener’s granulomatosis

## Abstract

Wegener's granulomatosis (WG) is the most frequent antineutrophil cytoplasmic antibody (ANCA)–associated vasculitis. It affects mainly the upper airways, lungs, and kidneys. Two forms are identified: systemic and limited. We describe three cases of limited WG diagnosed during a 7-year period. Our aim is to report three localized forms of WG and to put emphasis on the necessity of differentiating localized from systemic forms because of their different prognoses and manner of management. Our study contained two men and one woman with a mean age of 43 years. All our patients were symptomatic and presented with nonspecific respiratory signs. The cANCA were positive in all patients. The imaging findings consisted of cavitary masses. The diagnosis was based on surgical lung biopsy in all cases. All patients were put on cyclophosphamide and prednisolone. Only one patient presented with renal complications after a 2-year follow-up period. The two other patients did not present complications after, respectively, 1 month and 1 year of follow-up. These case reports put emphasis on a rare form of WG, the limited form. The low number of patients, due to the rarity of this disease, does not allow us to delineate the characteristics and the differences between this form and the systemic form, but we highlight the necessity of future investigations in order to explore the pathogenesis, therapeutic, and prognosis differences between these two subsets.

## INTRODUCTION

Systemic vasculitis diseases are rare, with an incidence of 20–100 cases/million and a prevalence of 150–450/million[1,2]. Wegener's granulomatosis (WG) is the most common antineutrophil cytoplasmic antibody (ANCA)–associated vasculitis. Its diagnosis is very challenging and its management depends on an early and accurate diagnosis.

## METHODS

The authors retrospectively reviewed the clinicopathologic records and biopsies of three patients diagnosed as having limited WG between 2002 and 2008. All the patients satisfied the American College of Rheumatology 1990 criteria for WG. All patients underwent an evaluation that included history taking and a thorough physical examination. Laboratory tests included white blood cell, hemoglobin, hematocrit, platelet count, C-reactive protein, erythrocyte sedimentation rate, ANCA, antinuclear antibody, and rheumatoid factor. Criteria for complete remission included the absence of active disease, complete resolution without signs of active inflammation, and the absence of systemic inflammatory disease.

## Observation No. 1

A 64-year-old man with a past medical history consisting of a renal lithiasis presented with chest pain and bloody sputum lasting for 2 months. Bronchoscopic endoscopy and cytology were normal. Chest X-ray showed a 7-cm heterogeneous opacity of the lower left lobe. CT scan revealed a basal excavated mass located in the left lung. Laboratory tests revealed the following: erythrocyte sedimentation rate, 27 mm/h, antinuclear factor present, and positive cANCA. Repeated urinalysis performed for active sediment and proteinuria was normal. Extemporaneous examination showed multiple granulomas as seen with a diagnosis of tuberculosis. Bacteriological tests were negative, especially for mycobacteria. A surgical biopsy was performed and histological examination showed an extensive irregular basophilic geographic necrosis with destructive vasculitis, scattered giant cells, and large numbers of inflammatory cells (Figs. 1B–D). The diagnosis of limited WG was retained. The patient was put on cyclophosphamide (2 mg/kg/day) for 4 weeks and prednisolone (1 mg/kg/day). After a 1-year follow-up period, he was asymptomatic. The chest X-ray and laboratory tests were normal.

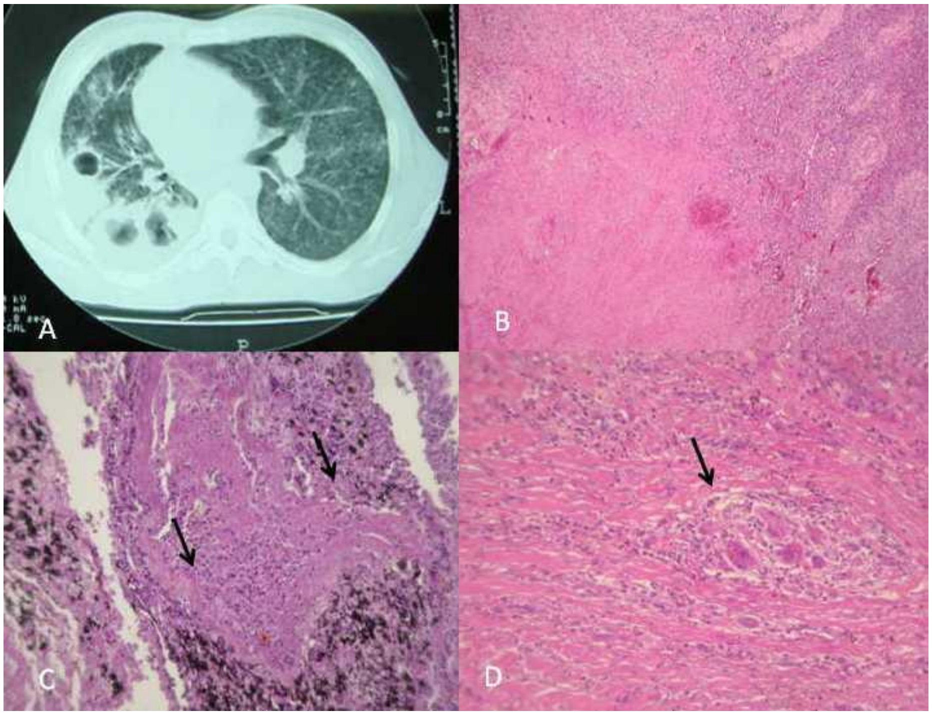

**FIGURE 1.** (A) CT scan of a patient showing bilateral cavitary masses. (B) Irregular "geographic" basophilic necrosis. (C) Destructive vasculitis (arrows identify remnants of elastic laminae). (D) A granulomatous inflammation with multinucleated giant cells (arrow).

## Observation No. 2

A 41-year-old woman, without a particular past medical history, presented with a dry cough, bloody sputum, and effort dyspnea for approximately 6 months. Chest X-ray showed peripheral bilateral excavated masses measuring from 1 to 2 cm. CT scan showed bilateral cavitary masses (Fig. 1A). Bronchial endoscopy and cytology were normal. Laboratory tests showed leukocytosis (7.8 × 109/L), anemia, thrombocytosis (platelet count of 300 × $10^9$/L), an elevated CRP, and positive pANCA. Bacteriological tests were performed to search for mycobacteria and aspergillus and were negative. A surgical lung biopsy through video-assisted thoracoscopic surgery was performed. Microscopic examination showed an ulcerated mucosa with mixed acute and chronic inflammation, and ill-defined granulomatous reaction surrounding necrosis and less often vasculitis of small vessels. The diagnosis of localized WG was retained and the patient was treated with standard therapy (daily cyclophosphamide and prednisolone). After a 1-month follow-up period, the patient presented a complete remission, but she was lost from view.

## Observation No. 3

A 26-year-old man presented for the exploration of a right chest pain, cough, and fever lasting for 10 months. A doctor of private practice put him on medical treatment that was not specified by the patient. Because of a deterioration of his general state, the patient presented to our consultation. Chest X-ray showed bilateral micronodular opacities. The bronchial fibroscopy was normal. The CT scan showed excavated masses of the right lung evoking atypical mycobacteriosis or tuberculosis. Laboratory tests showed leukocytosis, anemia (hemoglobin = 12.0 g/dL), a normal platelet rate, an elevated CRP, an increased sedimentation rate, and a normal renal function. The cANCA were negative. Bacteriological tests were negative for typical and atypical mycobacteria. A surgical biopsy was performed and revealed a pulmonary parenchyma with multiple epithelioid granulomatomas surrounding geographic necrosis. These lesions were extravascular, but similar changes were observed in the vessels, including fibrinoid degeneration of vessel walls and granulomatous vasculitis. The diagnosis of limited WG was retained and the patient was treated with daily cyclophosphamide and prednisolone. His general state improved for 2 years, then he presented with a renal involvement revealed by laboratory tests.

## DISCUSSION

WG was first described by Klinger in 1931, followed by other investigators, including Rossle in 1933, Wegener in 1936 and 1939, and Ringertz in 1947[3]. WG is a multifocal inflammatory disease that most often affects the upper and lower respiratory tracts and the kidneys[4]. In the mid-1960s, limited forms were recognized. Specific definitions of these forms have varied in the literature, but the current consensus among vasculitis experts is that limited disease, in contrast to the severe form, includes manifestations of WG that pose no immediate threat to either the patient's life or the function of a vital organ[5,6]. According to this definition, our three patients are considered to have limited illness. Many authors describe a male predominance in severe disease, whereas limited illness seems to be more common in women[4]. We report the cases of two men and one woman. This finding is not compatible with the literature data, but the small number of cases does not allow us to make conclusions. Few reports attempted to compare the disease features of patients with limited WG and those with severe WG. Earlier reports emphasized that limited WG may evolve into severe disease, implying that these two states are part of the same disease spectrum. They attributed the lack of progression in some cases to the treatment. Other authors advocate the fact that they are two different diseases. This is due to the different pattern of organ involvement between the two groups and the more frequent relapses in patients with limited disease. Besides, patients whose disease features were consistent with limited disease at the time of their original diagnosis tended to remain true to their initial presentation. Our third patient presented ultimate renal localizations, but this event can be observed in the course of a limited form[4]. A number of studies in the literature suggest a possible pathophysiologic basis for the differences between the two forms. In an examination of nasal biopsy, Müller and coworkers noted differences in cytokine expression across the localized and generalized disease subsets[7]. Pulmonary involvement is one of the cardinal features of WG. It occurs in 87% during its course and it is observed only in 45% of patients at presentation[8]. Our three patients presented pulmonary manifestations at the onset of the disease. The clinical features of pulmonary involvement are nonspecific comprising cough, dyspnea, hemoptysia, and bloody sputum[9]. Fever and weight loss may be reported at the onset of the disease[8]. In limited forms, the pulmonary lesions are usually multiple and bilateral, often simulate primary or metastatic neoplasms of the lung, but may suggest infectious granulomatous disease. A tendency to involve the lower portions of the lung is useful in differentiating between these lesions and those of tuberculosis[10]. According to these findings, our patients presented either bilateral lesions or a unilateral mass of the lower part of the lung. The diagnosis of WG remains clinicopathologic even if some authors have suggested that the classification of vasculitis should be based on immunologic findings rather than on pathologic patterns. cANCA positivity is characteristic of WG and is found in 90% or more of patients with disseminated disease. Occasional histologically typical cases, like our second patient, show pANCA positivity (about 5% of cases); 15% are negative and this is more common in localized disease. False-positive cANCA tests (in infection and collagen vascular diseases) are occasionally encountered. pANCA is less specific than cANCA. The positive predictive value of ANCA testing is from 19 to 66%[8]. In spite of the utility of ANCA, their presence is not required to make a diagnosis of WG by either the American College of Rheumatology or the Chapel Hill Consensus Conference definitions[1]. Surgical lung biopsy gives a definitive diagnosis in the clear majority of cases[9]. The pulmonary lesions often resemble "white infarcts" grossly. This appearance is so characteristic as to suggest the nature of the lesion on gross examination. Cavitation occurs in approximately half of the cases[10]. Three components are identified: vasculitis of both arteries and veins, geographic zones of basophilic necrosis, and an inflammatory background. These components determine various patterns, such as alveolar hemorrhage, organizing pneumonia, bronchocentric lesions, pleural involvement, sclerotic nodules, nonspecific cellular interstitial infiltrates, etc.[8]. Differential diagnoses include infection, inflammatory bowel disease–related lung disease, and the other ANCA-associated vasculitides. The presence of bizarre reticuloendothelial cells may suggest Hodgkin's disease, but angiitis is a distinguishing feature. WG is characterized by a frequent relapse estimated by some authors to be 52%[8]. This particularity resulted in a two-part model treatment: an induction phase and a maintenance phase. Treatment is based on aggressive immunosuppression that is more intensive in the first phase[11]. The classical treatment for WG includes prednisolone (1 mg/kg/day), accompanied by cyclophosphamide (2–3 mg/kg:day) for 4–6 weeks; the cyclophosphamide dose being adjusted according to the lymphocyte count (which should be maintained at approximately 1,000 cells/$mm^3$) and the prednisone being tapered over the subsequent 6 months[12] The use of cyclophosphamide and corticosteroids has remained the first-line therapy. Novel agents are also available, in refractory cases such as tumor necrosis factor-α inhibitor, anti-CD20 therapy (rituximab), but evidence from controlled trials is still lacking[11]. In terms of the current standard of care, the distinction between limited and severe disease is important, the former requiring less-toxic alternative regimens consisting of methotrexate and glucocorticoids, and the latter requiring prompt institution of an aggressive therapeutic regimen including cyclophosphamide and glucocorticoids[4]. Limited forms of WG seem to have a better prognosis than systemic forms in term of survival, but there is no accurate percentages related to them.

## CONCLUSION

These case reports put emphasis on a rare form of WG, the limited form. The low number of patients, due to the rarity of this disease, does not allow us to delineate the characteristics and the differences between this form and the systemic form, but we highlight the necessity of future investigations in order to explore the pathogenesis, therapeutic, and prognosis differences between these two subsets.
